# A deep learning model for the classification of indeterminate lung carcinoma in biopsy whole slide images

**DOI:** 10.1038/s41598-021-87644-7

**Published:** 2021-04-14

**Authors:** Fahdi Kanavati, Gouji Toyokawa, Seiya Momosaki, Hiroaki Takeoka, Masaki Okamoto, Koji Yamazaki, Sadanori Takeo, Osamu Iizuka, Masayuki Tsuneki

**Affiliations:** 1 Medmain Research, Medmain Inc., Fukuoka, 810-0042 Japan; 2 Department of Thoracic Surgery, Clinical Research Institute, National Hospital Organization, Kyushu Medical Center, Fukuoka, 810-8563 Japan; 3 Department of Pathology, Clinical Research Institute, National Hospital Organization, Kyushu Medical Center, Fukuoka, 810-8563 Japan; 4 Department of Respiratory Medicine, Clinical Research Institute, National Hospital Organization, Kyushu Medical Center, Fukuoka, 810-8563 Japan; 5 Medmain Inc., Fukuoka, 810-0042 Japan

**Keywords:** Computer science, Lung cancer

## Abstract

The differentiation between major histological types of lung cancer, such as adenocarcinoma (ADC), squamous cell carcinoma (SCC), and small-cell lung cancer (SCLC) is of crucial importance for determining optimum cancer treatment. Hematoxylin and Eosin (H&E)-stained slides of small transbronchial lung biopsy (TBLB) are one of the primary sources for making a diagnosis; however, a subset of cases present a challenge for pathologists to diagnose from H&E-stained slides alone, and these either require further immunohistochemistry or are deferred to surgical resection for definitive diagnosis. We trained a deep learning model to classify H&E-stained Whole Slide Images of TBLB specimens into ADC, SCC, SCLC, and non-neoplastic using a training set of 579 WSIs. The trained model was capable of classifying an independent test set of 83 challenging indeterminate cases with a receiver operator curve area under the curve (AUC) of 0.99. We further evaluated the model on four independent test sets—one TBLB and three surgical, with combined total of 2407 WSIs—demonstrating highly promising results with AUCs ranging from 0.94 to 0.99.

## Introduction

Lung cancer is the leading cause of cancer-related death in many countries, and its prognosis still remains unsatisfactory^1^. Although surgical resection remains the primary treatment option for patients with lung cancer, novel therapeutic options, such as molecular-targeted therapy and immunotherapy, have greatly improved the prognosis of patients with advanced lung cancer, as such therapies are based on the given the tumour genetics and micro-environment. Treatment approaches differ substantially based on the type of malignant tumour; therefore, accurate histopathological diagnosis is of great importance^2,3^.

For therapeutic purposes, lung carcinomas fall into two major groups: small cell lung cancer (SCLC) and non-small cell lung cancer (NSCLC). The latter category is mainly composed of adenocarcinoma (ADC) and squamous cell carcinoma (SCC)^3^. The carcinoma subtypes differ in their malignant behaviours and responses to treatments, so histopathological typing is clinically of considerable importance. Pathologists typically diagnose lung cancers based on Hematoxylin and Eosin (H&E) staining of small transbronchial lung biopsies (TBLB)^4,5^; however, on some TBLB specimens, H&E staining is not enough to reach a diagnosis, and pathologists frequently use immunohistochemical (IHC) staining of the specimen to confirm a definitive diagnosis^6^.

The pathologists’ typical workflow for lung carcinoma diagnosis is as follows: they start by visually examining the H&E-stained TBLB specimen. If the specimen contains well-differentiated cancer cells, then the pathologists can easily classify the cancer subtype; however, if the specimen solely contains poorly-differentiated cancer cells, then the pathologists find that distinguishing between subtypes is particularly challenging. The incidence of such cases is non-negligible at about 28% of the population^7^. In order to confirm the diagnosis for a challenging specimen, pathologists tend to perform further investigations via IHC stainings^8–11^ and/or deferred to surgical resections. It can be difficult to subclassify poorly differentiated carcinoma as ADC or SCC^11^. This is because well-differentiated SCC shows prominent keratinization throughout the cells whereas poorly-differentiated carcinomas which lack keratinization and/or intercellular bridges require careful scrutiny. Histopathological diagnosis from surgical resection specimens are more accurate. This is due to the considerable variation of cancer cells in the large tissue area of surgical specimens, and so there is a high likelihood of the presence of well-differentiated carcinoma cells which are easier to classify.

Whole slide images (WSI) are the digitised counterparts of glass slides and are obtained at magnifications up to x40, resulting in massive high-resolution images with billions of pixels. Due to their size, some WSIs can be tedious to visually inspect exhaustively. This is where computational pathology comes in as a tool to assist pathologists, where image analysis methods based on machine and deep learning have found many successful applications^12–25^. In particular, for lung histopathology, deep learning has been applied primarily on lung surgical resections to classify lung carcinoma and/or subtypes as well as predicting mutations^12,18,19,26,27^. Coudray et al.^19^ evaluated their deep learning model that was trained on surgical resection WSIs obtained from The Cancer Genome Atlas (TCGA)^28^ on a test set of biopsies (n = 102, 51 ADC, 51 SCC) achieving receiver operator curve (ROC) area under the curves (AUC) of 0.871 and 0.928 for ADC and SCC, respectively. However, the algorithm had a relatively poor performance on the poorly-differentiated biopsy specimens (n=34), where the ROC AUCs were 0.809 (CI 0.639–0.940) and 0.822 (CI, 0.658–0.951) for ADC and SCC, respectively.

In this study, we demonstrate that a deep learning model, consisting of a convolutional neural network (CNN) and a recurrent neural network (RNN), can be trained to predict lung carcinoma subtypes in indeterminate TBLB specimens. These are specimens consisting of poorly-differentiated carcinomas that pathologists typically find challenging to diagnose from H&E-stained slides alone, and these either require further immunohistochemistry or are deferred to surgical resection for definitive diagnosis. We trained a deep learning model to classify WSIs into ADC, SCC, SCLC, and non-neoplastic using a training set of 579 WSIs of TBLB specimens. We evaluated the model on a test set of indeterminate specimens (n=83) achieving an AUC of 0.99. We then evaluated the model on four additional test sets of which one was TBLB specimens (n=502) and three were surgical resections obtained from different medical institutions (combined total of 2,407). These results suggest that computational algorithms might be useful as adjunct diagnostic tests to help with sub-classification of primary lung neoplasms.

## Results

### A deep learning model for lung subtype carcinoma TBLB WSI classification

**Figure 1:**
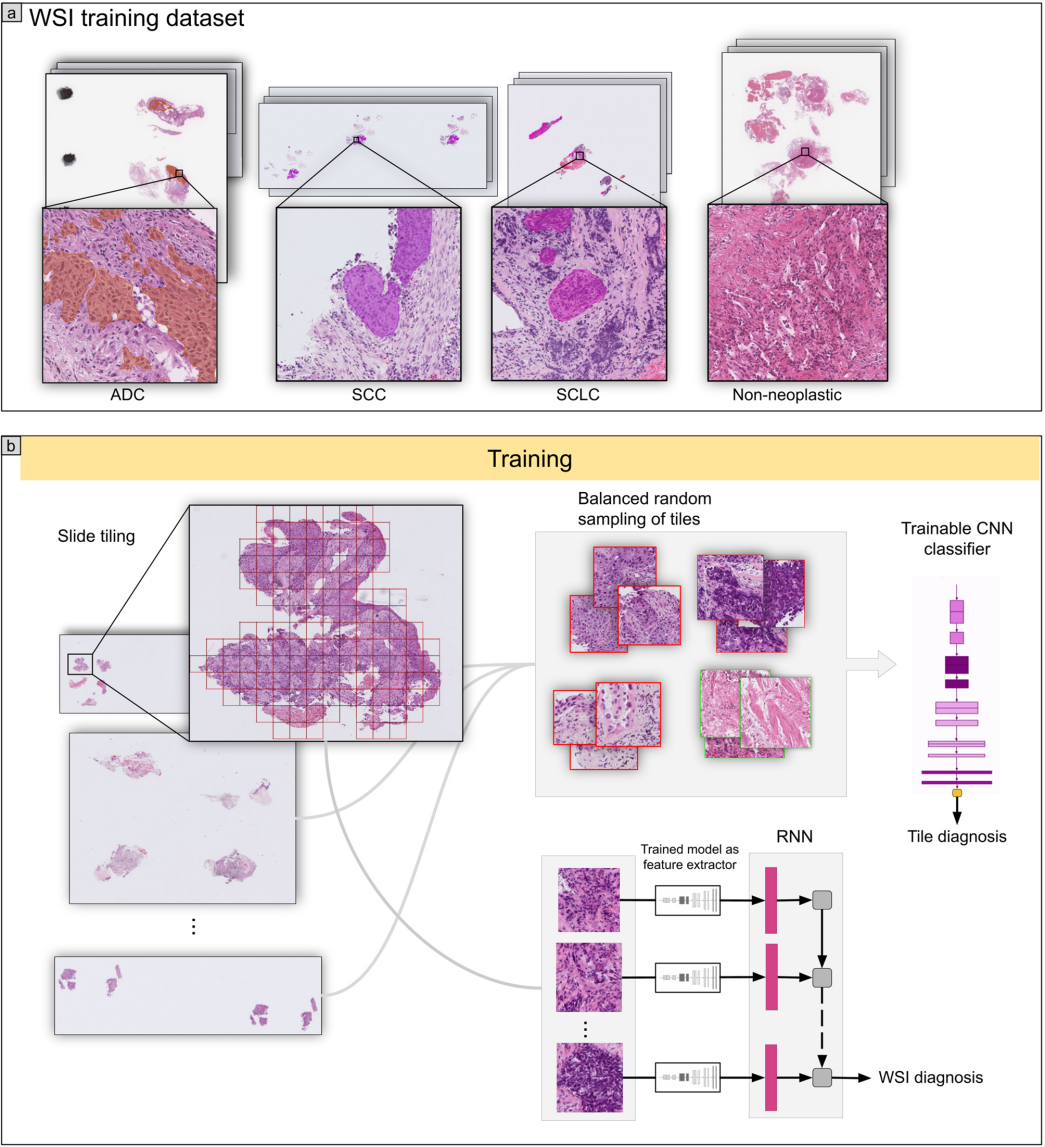
(**a**) shows representative WSIs from the training set for each of the four labels: ADC, SCC, SCLC, and non-neoplastic. (**b**) shows a higher level overview of the training method, where tiles are randomly sampled, in a balanced manner, from the training WSIs and provided as input to train the CNN model. The CNN model is then used as a feature extractor by passing in the outputs from the penultimate layer of the CNN as input to the RNN model. All the tiles from a given WSI are fed into the RNN to provide a final WSI diagnosis.

**Figure 2:**
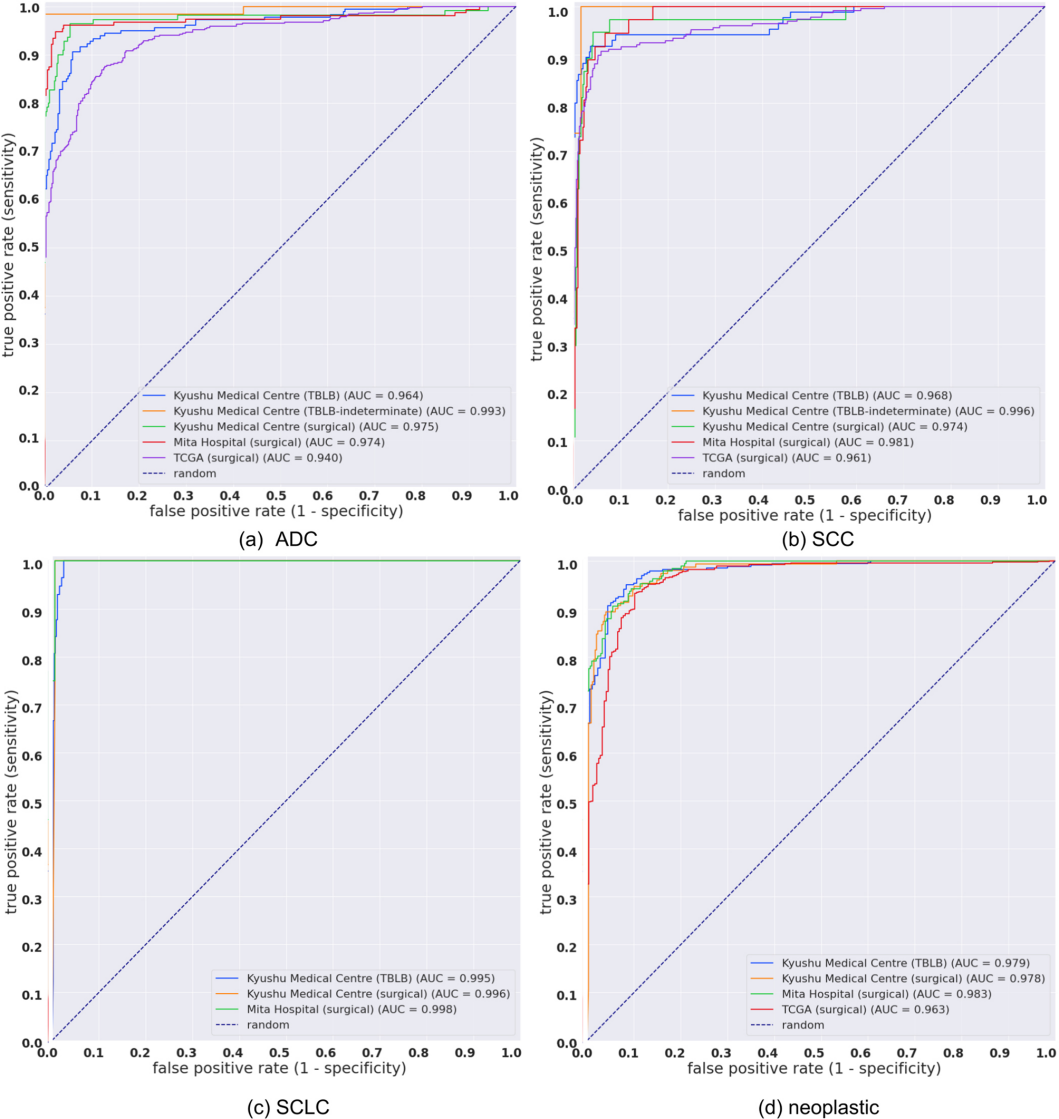
ROC curves for the five tests sets for each output label (**a**) ADC, (**b**) SCC, (**c**) SCLC. The neoplastic label (**d**) is a grouping of ADC, SCC, and SCLC and effectively evaluates the classification of carcinoma regardless of subtype.

The aim of this study was to develop a deep learning model to classify lung carcinoma subtypes from WSIs of TBLB specimens, in particular with the aim of evaluating it on a challenging test set of indeterminate cases. These are cases for which diagnoses were confirmed via IHC stainings and/or after surgical resections. To this end, we used a training set consisting of 579 WSIs of TBLB specimens from Kyushu Medical Centre, and we used it to train a deep learning model to classify carcinoma subtypes (ADC, SCC, and SCLC) and non-neoplastic with a pipeline as outlined in Fig. 1. The training set consisted of 534 WSIs for which the diagnosis was determined by pathologists from visual inspection of the H&E slides and of 45 WSIs of indeterminate cases. We then evaluated the models on five independent test sets (see Table 2 for distribution of WSIs). For each test set, we computed the ROC AUC of two approaches for obtaining the WSI diagnosis: (1) our main method of using a CNN model to obtain tile predictions followed by an RNN model to aggregate the tile predictions into a single WSI diagnosis, and (2) using the CNN model only and max-pooling the probabilities of all the tiles to obtain a WSI diagnosis (see Methods for details). These results are summarised in Table 1. Figure 2 shows the ROC curves of on all the test sets for each label from using the main method only.

**Table 1:** ROC AUCs for ADC, SCC, and SCLC computed on the test sets in which they are present, with the WSI diagnosis obtained with either the RNN model or max-pooling. The ROC AUCs were also computed for the neoplastic label by grouping ADC, SCC, and SCLC.

		ROC AUC w/RNN	ROC AUC w/max-pool
ADC	Kyushu Medical Centre (TBLB)	0.964 (0.942–0.978)	0.922 (0.901–0.944)
	Kyushu Medical Centre (TBLB-indeterminate)	0.993 (0.971—1.0)	0.814 (0.684-0.891)
	Kyushu Medical Centre (surgical)	0.975 (0.95—0.995)	0.97 (0.954-0.984)
	Mita Hospital (surgical)	0.974 (0.951–0.993)	0.987 (0.978–0.995)
	TCGA (surgical)	0.94 (0.923–0.952)	0.822 (0.798–0.848)
SCC	Kyushu Medical Centre (TBLB)	0.968 (0.941–0.99)	0.974 (0.959–0.987)
	Kyushu Medical Centre (TBLB-indeterminate)	0.996 (0.981–1.0)	0.989 (0.957–1.0)
	Kyushu Medical Centre (surgical)	0.974 (0.937–0.994)	0.985 (0.975–0.994)
	Mita Hospital (surgical)	0.981 (0.966–0.993)	0.979 (0.965–0.994)
	TCGA (surgical)	0.961 (0.944–0.976)	0.959 (0.944–0.97)
SCLC	Kyushu Medical Centre (TBLB)	0.995 (0.99–0.999)	0.994 (0.998–0.999)
	Kyushu Medical Centre (surgical)	0.996 (0.991–1.0)	0.995 (0.991–1.0)
	Mita Hospital (surgical)	0.999 (0.993–1.0)	0.999 (0.992–1.0)
Neoplastic	Kyushu Medical Centre (TBLB)	0.979 (0.968–0.988)	0.992 (0.987–0.997)
	Kyushu Medical Centre (surgical)	0.978 (0.967–0.989)	0.988 (0.979—0.995)
	Mita Hospital (surgical)	0.983 (0.974–0.99)	0.995 (0.991–0.999)
	TCGA (surgical)	0.963 (0.947–0.975)	0.983 (0.976–0.99)

### Deep learning model can distinguish between ADC and SCC on indeterminate TBLB test set

**Figure 3:**
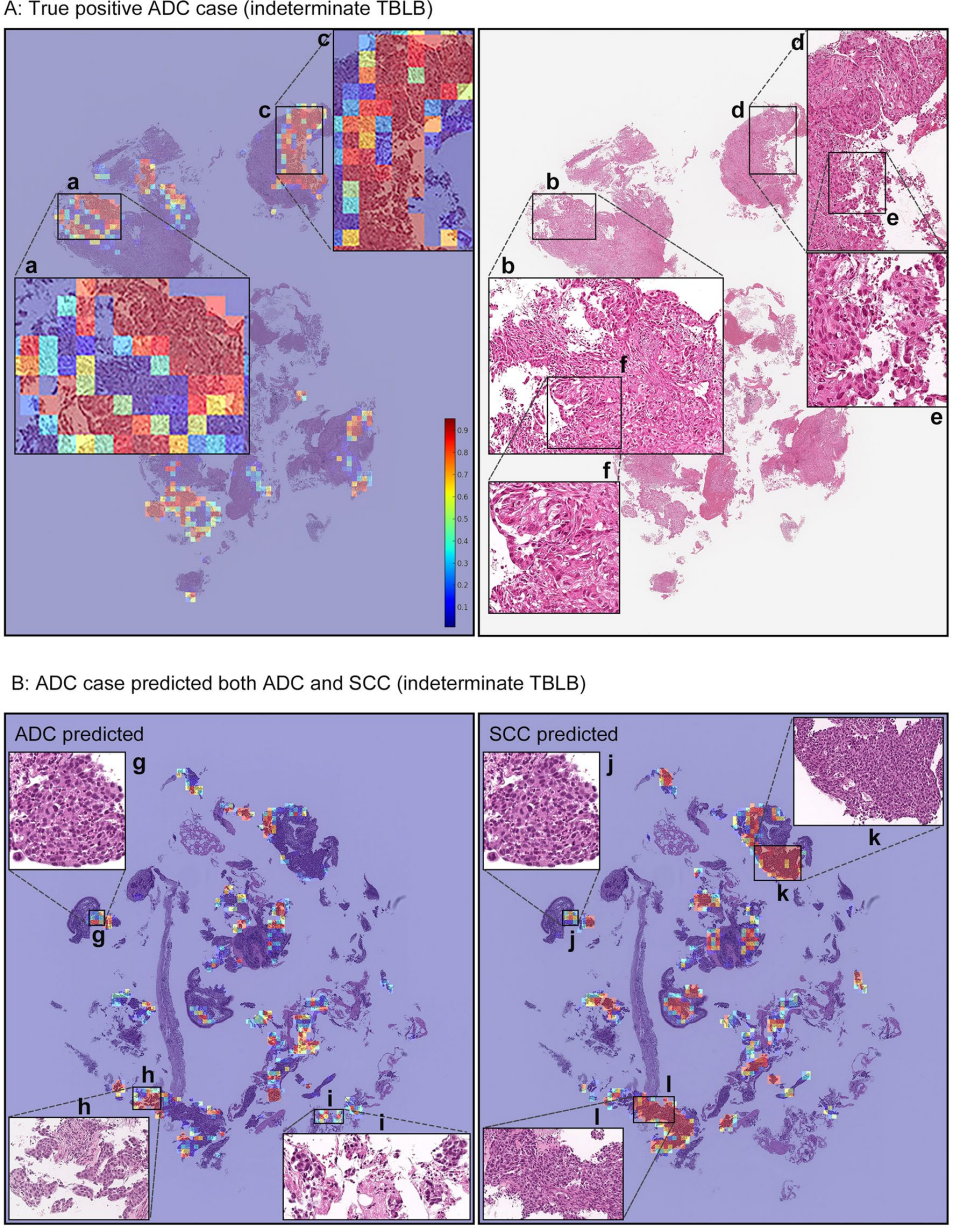
(**A**) shows a true positive ADC case (ADC-046) from the indeterminate TBLB test set. Heatmap images (**a**) and (**c**) show true positive predictions of ADC cells, and they correspond respectively to (**b**) and (**d**). The high magnification (**e**) and (**f**) subimages show spindle shaped and poorly differentiated morphology. Pathologists found it challenging to distinguish between ADC and SCC based on H&E histology alone. (**B**) shows a case (ADC-009) that was predicted as indeterminate, with the model showing strong predictions for both ADC and SCC. The (**g**) and (**j**) areas are almost overlapped, and based on the histology it is poorly differentiated and is impossible to decide between ADC and SCC. (**k**) and (**l**), and (**h**) and (**i**) have similar morphologies to poorly differentiated SCC and ADC, respectively, and the model strongly predicted them as such, respectively. In the heatmap colour spectrum, red indicates high probability, blue indicates low.

**Figure 4:**
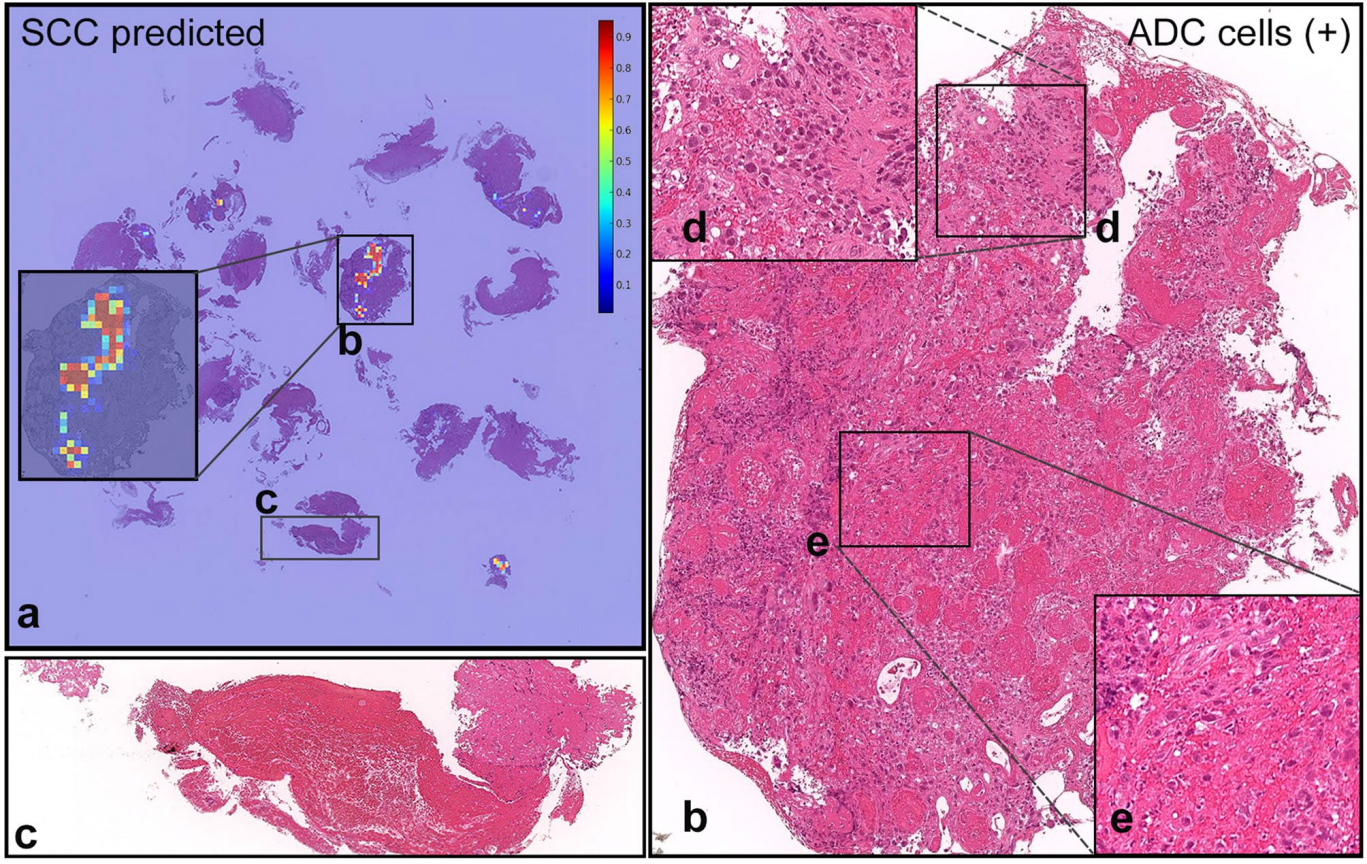
The true diagnosis of this case (ADC-029) is ADC; however, it was predicted as SCC. (**a**) shows probability heatmap for SCC. The ADC cells highlighted in (**d**) and (**e**) from the (**b**) fragment are floating in a single-cell manner within necrotic tissue, which is potentially the source of confusion for the model. (**c**) shows non-neoplastic necrotic tissue without any cancer cells. In the heatmap colour spectrum, red indicates high probability, blue indicates low.

We applied the model on an indeterminate test set consisting of 83 H&E-stained WSIs of TBLB specimens from Kyushu Medical Centre. Pathologists determined the final diagnoses either via IHC and/or examination of surgically resected specimens. This resulted in confirmed diagnoses of 64 ADCs and 19 SCCs (Table 2). Table 3 presents detailed case by case findings from the IHC and/or after surgical resection as well as the predictions from our deep learning model. Overall, the model (CNN+RNN) achieved an AUC of 0.993 (CI 0.971-1.0) and 0.996 (0.981-1.0) for ADC and SCC, respectively. Figures 3 and 4 show probability heatmaps for a representative true positive ADC prediction as well as two false positive predictions of SCC.

**Table 2:** Distribution of subtype labels in the training set and the five test sets.

		ADC	SCC	SCLC	Non-neoplastic	Total
Training	Kyushu Medical Centre (TBLB)	76	48	17	393	534
	Kyushu Medical Centre (TBLB-indeterminate)	28	17	0	0	45
Validation	Kyushu Medical Centre (TBLB)	18	7	4	30	59
Test	Kyushu Medical Centre (TBLB)	180	85	57	180	502
	Kyusyu Medical Centre (TBLB-indeterminate)	64	19	0	0	83
	Kyushu Medical Centre (surgical)	110	37	4	349	500
	Mita Hospital (surgical)	152	36	4	308	500
	TCGA (surgical)	433	226	0	246	905
Total		1061	475	86	1506	3128

**Table 3:** Detailed IHC, surgical, and AI prediction for the 83 cases in the indeterminate test set.

Case No.	Immunohistochemistry (IHC)	Surgical specimen diagnosis	TBLB-final diagnosis	TBLB-AI prediction
ADC-001		ADC	ADC	ADC
ADC-002		ADC	ADC	ADC
ADC-003	TTF1 (+), Napsin-A (-), p40 (-), CK5/6 (-)	ADC	ADC	ADC
ADC-004		ADC	ADC	ADC
ADC-005	TTF1 (+)	No surgery	ADC	ADC
ADC-006		ADC	ADC	ADC
ADC-007		ADC	ADC	ADC
ADC-008		ADC	ADC	ADC
ADC-009	TTF1 (+), p40 (-)	No surgery	ADC	**SCC**, ADC
ADC-010		ADC	ADC	ADC
ADC-011		ADC	ADC	ADC
ADC-012		ADC	ADC	ADC
ADC-013		ADC	ADC	ADC
ADC-014	TTF1 (+)	No surgery	ADC	ADC
ADC-015		ADC	ADC	ADC
ADC-016	TTF1 (+), Napsin-A (+), p40 (-), CK5/6 (-)	No surgery	ADC	ADC
ADC-017		ADC	ADC	ADC
ADC-018	TTF1 (+), p40 (-)	No surgery	ADC	ADC
ADC-019		ADC	ADC	ADC
ADC-020	TTF1 (+), p40 (-), CK5/6 (-)	No surgery	ADC	ADC
ADC-021	TTF1 (+), CK20 (-), p63 (-), Uroplakin II (-), Thrombomodulin (-)	ADC	ADC	ADC
ADC-022		ADC	ADC	ADC
ADC-023		ADC	ADC	ADC
ADC-024		ADC	ADC	ADC
ADC-025	TTF1 (+), Napsin-A (+), p40 (-), CK5/6 (-)	No surgery	ADC	ADC
ADC-026		ADC	ADC	ADC
ADC-027		ADC	ADC	ADC
ADC-028	TTF1 (+), Napsin-A (+), p40 (-), CK5/6 (-)	No surgery	ADC	ADC
ADC-029	TTF1 (+), SP-A (-), p40 (-), CK5/6 (-)	No surgery	ADC	**SCC**
ADC-030		ADC	ADC	ADC
ADC-031		ADC	ADC	ADC
ADC-032		ADC	ADC	ADC
ADC-033		ADC	ADC	ADC
ADC-034		ADC	ADC	ADC
ADC-035		ADC	ADC	ADC
ADC-036	TTF1 (+), CEA (+), SP-A (-), p40 (-), CK5/6 (-), p63 (-)	No surgery	ADC	ADC
ADC-037		ADC	ADC	ADC
ADC-038		ADC	ADC	ADC
ADC-039		ADC	ADC	ADC
ADC-040		ADC	ADC	ADC
ADC-041		ADC	ADC	ADC
ADC-042		ADC	ADC	ADC
ADC-043		ADC	ADC	ADC
ADC-044	TTF1 (+), Napsin-A (+)	No surgery	ADC	ADC
ADC-045	TTF1 (+), SP-A (+)	No surgery	ADC	ADC
ADC-046	TTF1 (+), SP-A (+), CEA (+), CK5/6 (-), p40 (-), p63 (-)	No surgery	ADC	ADC
ADC-047	TTF1 (+), SP-A (+), CEA (+), CK5/6 (-), p40 (-), p63 (-)	ADC	ADC	ADC
ADC-048		ADC	ADC	ADC
ADC-049	CEA (+), CK5/6 (-), p40 (-), p63 (-)	No surgery	ADC	ADC
ADC-050		ADC	ADC	ADC
ADC-051	CEA (+), CK5/6 (-), p40 (-), p63 (-)	ADC	ADC	ADC
ADC-052		ADC	ADC	ADC
ADC-053	CK7 (+), TTF-1 (+), SP-A (+), MUC1 (+)	ADC	ADC	ADC
ADC-054	TTF1 (+), MUC1 (+), MUC2 (-), MUC5AC (+), MUC6 (+)	ADC	ADC	ADC
ADC-055		ADC	ADC	ADC
ADC-056		ADC	ADC	ADC
ADC-057		ADC	ADC	ADC
ADC-058		ADC	ADC	ADC
ADC-059	AE1/AE3 (+), TTF1 (+), Vimentin (-), LCA (-)	ADC	ADC	ADC
ADC-060		ADC	ADC	ADC
ADC-061		ADC	ADC	ADC
ADC-062		ADC	ADC	ADC
ADC-063		ADC	ADC	ADC
ADC-064		ADC	ADC	ADC
SCC-001	TTF1 (-), SP-A (-), CK5/6 (+), p63 (+), p40 (+), CEA (-)	SCC	SCC	SCC
SCC-002	TTF1 (-), SP-A (-), CK5/6 (+), p63 (+), p40 (+), CEA (+), involcrin (+)	No surgery	SCC	SCC
SCC-003		SCC	SCC	SCC
SCC-004		SCC	SCC	SCC
SCC-005		SCC	SCC	SCC
SCC-006	CK5/6 (+), p63 (+), p40 (+), CEA (-), CD56 (-), Synaptophysin (-), Chromogranin A (-)	No surgery	SCC	SCC
SCC-007	CK5/6+, CK7+, p63+, TTF-1-, SP-A-	No surgery	SCC	SCC
SCC-008		SCC	SCC	SCC
SCC-009	CK5/6 (+),p63 (+), CEA (+), Involucrine (+), TTF1 (-)	No surgery	SCC	SCC
SCC-010		SCC	SCC	SCC
SCC-011		SCC	SCC	SCC
SCC-012	CK14 (+), CK7 (+), CK5/6 (+), p63 (+), TTF1 (-), SP-A (-), ER (-), PgR (-)	No surgery	SCC	SCC
SCC-013		SCC	SCC	SCC
SCC-014		SCC	SCC	SCC
SCC-015	TTF1 (-), SP-A (-), p63 (+), CK7 (-)	SCC	SCC	SCC
SCC-016		SCC	SCC	SCC
SCC-017		SCC	SCC	SCC
SCC-018	TTF1 (-)	No surgery	SCC	SCC
SCC-019	TTF1 (-)	No surgery	SCC	SCC

### Deep learning model can classify subtypes on TBLB test set

We applied our model on a test set consisting of 502 WSIs of TBLB specimens from Kyushu Medical Centre, the same source as the training set. Pathologists were able to diagnose these cases from visual inspection of the H&E-stained slides alone. The model (CNN+RNN) achieved an ROC AUC of 0.964 (CI 0.942–0.978), 0.968 (CI 0.941–0.99), and 0.995 (CI 0.99–0.999) for ADC, SCC, and SCLC, respectively. In addition, when grouping all the labels to perform a classification of neoplastic vs non-neoplastic, the model achieved an AUC of 0.979 (CI 0.968–0.988).

### Deep learning model can predict carcinomas on practical surgical sections

**Figure 5:**
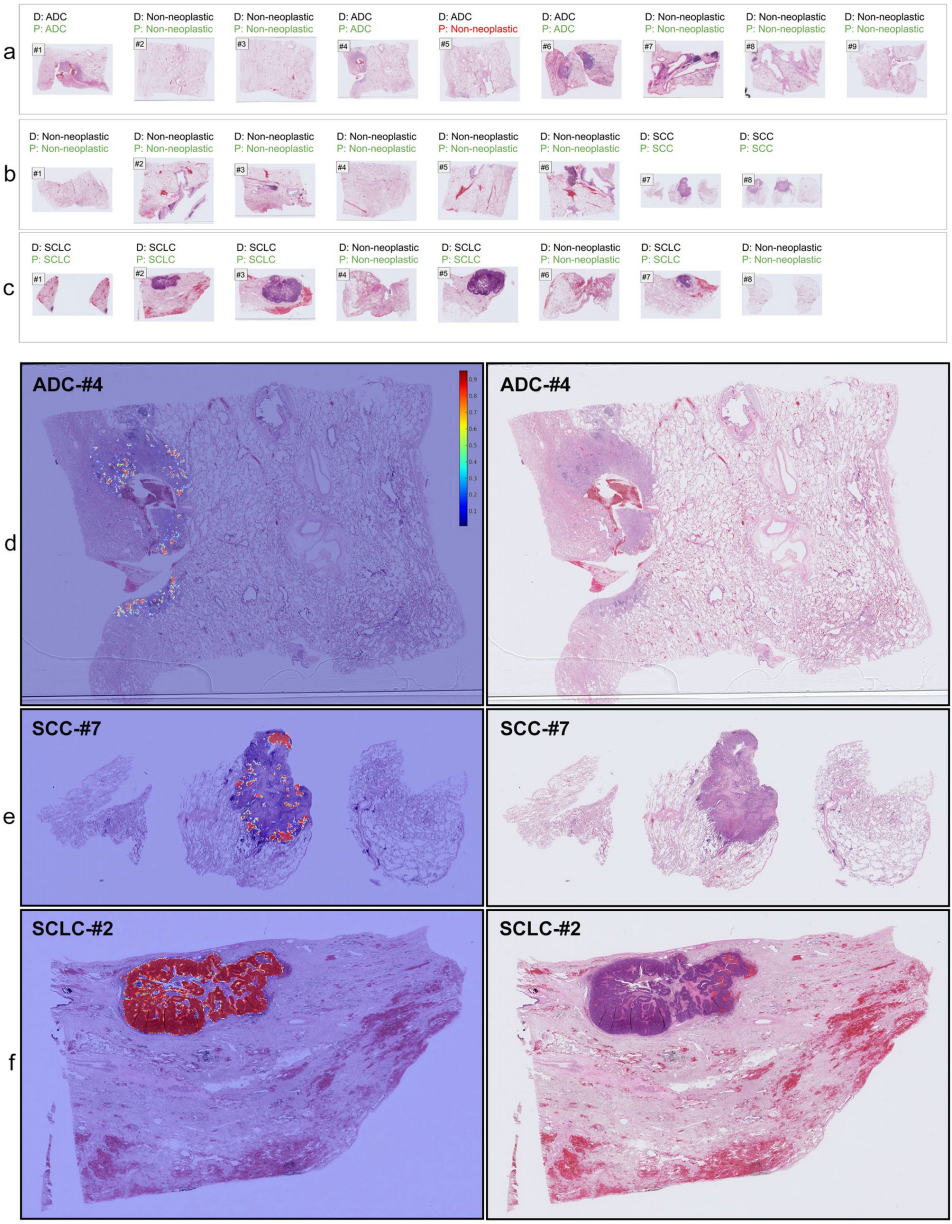
(**a**–**c**) show representative surgical serial sections for ADC (#1-#9), SCC (#1-#8), and SCLC (#1-#8), respectively, and their associated diagnosis (**D**) and prediction (**P**) by our model. (**a**) #5 is a false negative prediction while all the rest are true positives. (**d**–**f**) show representative true positive probability heatmaps for ADC (**d**), SCC (**e**), and SCLC (**f**), respectively. Histopathologically, all the detected areas correspond to cancer cells. In the heatmap colour spectrum, red indicates high probability, blue indicates low.

Even though we trained the model using only TBLB specimens, we tested the model on surgically-resected lung specimens to further evaluate its generalisation. The primary difference between biopsies and surgical resections is the sheer size of the tissue area. To this end, we obtained a test set of surgical specimens (n=500) from Kyushu Medical Centre—the same source as the training set—and two test sets from external sources: Mita Hospital (n=500) and TCGA (n=905). For the Kyushu Medical Centre test set, the model (CNN+RNN) achieved an AUC of 0.975 (0.95-0.995), 0.974 (0.937-0.994), and 0.996 (0.991-1.0) for ADC, SCC, and SCLC, respectively. For the Mita Hospital test set, the model (CNN+RNN) achieved an AUC of 0.974 (0.951-0.993), 0.981 (0.966-0.993), and 0.999 (0.993-1.0), respectively. And finally, for the TCGA test set, the model (CNN+RNN) achieved an AUC of 0.940 (CI, 0.923-0.952), 0.961 (CI, 0.944-0.976) for ADC and SCC, respectively. The TCGA test set did not contain any SCLC cases (see Table 2). Figure 5 (a–c) show serial sections of surgical specimens containing ADC, SCC, and SCLC with their correct diagnoses and the predicted WSI diagnoses, while Fig. 5 (d–f) show representative heatmap prediction outputs for ADC, SCC, and SCLC.

## Discussion

Our study has demonstrated that a deep learning model, composed of a CNN and an RNN, can be trained to predict lung carcinoma subtypes even on H&E-stained TBLB specimens which solely contain poorly-differentiated carcinomas. These are specimens that are more difficult to diagnose by pathologists. The model almost perfectly classified the indeterminate test set with an AUC of 0.99. The AUCs for previous reported results for indeterminate cases (n=34)^19^ of ADC and SCC were 0.809 (CI 0.639–0.940) and 0.822 (CI, 0.658–0.951). In addition, we evaluated the model on one test set of TBLB specimens (n=502) and three test sets of surgical specimens (n=500,500,905) achieving high ROC AUCs in the range of 0.94 to 0.99.

The model performed the least well for ADC on the TCGA test set. The primary reason for this could be due to the difference in image type and quality between the training set and the TCGA test set. The TCGA test set consisted of a mix of formalin fixed paraffin embedded (FFPE) surgical specimens as well as frozen section specimens. In addition, there was a large variation in appearances present due to crushed/scratched tissues, bubbles, and non-consistent staining colour. On the other hand, the training TBLB set consisted only of FFPE specimens and were all obtained with a consistent quality. Training on specimens that have similar characteristics to WSIs in the TCGA test set will most likely lead to an improvement in performance on TCGA.

We have found that using an RNN model to obtain the WSI diagnosis as opposed to using a simple max-pooling was highly beneficial for distinguishing between ADC and SCC in the presence of poorly-differentiated cancer cells. For instance, on the indeterminate test set, there is a large difference in AUC (0.814 vs 0.993). A visual inspection of the cases in the test set revealed that, when using the max-pooling approach, a significant number of SCC cases were also being predicted as ADC, and the areas being predicted as ADC in the SCC cases were tiles with exclusively poorly-differentiated cancer cells. A primary cause of this is most likely due to the way our training dataset was annotated. An ADC case that had both well-differentiated and poorly-differentiated cancer cells present had all the cells labelled as ADC, similarly for SCC. When encountering a poorly differentiated cell amongst well-differentiated cells then the pathologists automatically labelled it the same label as the surrounding well-differentiated cells. However, if those poorly differentiated cells were looked at in isolation without the bias of information from the well-differentiated cells, then for some of them, there would have been ambiguity in what their label should be. In addition, our training dataset contained more ADC than SCC cases; therefore, when viewed in isolation, only within the scope of a 224x224 tile, this skewed the prediction of poorly-differentiated cells towards ADC. On the other hand, the RNN model allows integrating information from all the tiles before making a WSI diagnosis. The RNN model significantly improved the distinction between ADC and SCC for cases with poorly-differentiated cancer cells resulting in a reduction in false positives; however, this led to a minor reduction in the overall neoplastic prediction for all test sets (e.g., Kyushu Medical Centre (TBLB) AUC reduced from 0.992 to 0.979; see 4th sub-row in Table 1). The RNN model corrected a large number of false positives, and, as a side effect, led to a minor increase in false negatives.

While we have not adopted the approach here, a hybrid approach could be used to minimise false negatives by first detecting all the neoplastic cases using the max-pooling approach, regardless of subtype, and then applying the RNN model as a second stage to attempt to distinguish between subtypes.

Figure 3B shows a case where the model had strong predictions for both ADC and SCC. A reinspection of this case by pathologists found that it was difficult to be able to conclusively decide between ADC and SCC for this case based on the H&E-stained slide alone. The true diagnosis of this case is ADC as confirmed by IHC. Figure 3A shows a probability heatmap of a true positive prediction of ADC on a case from the indeterminate TBLB specimen. Similarly, a reinspection by pathologists found this case particularly challenging to decide between ADC and SCC. Despite that, our model was able to predict the correct diagnosis as confirmed via IHC.

The performance of the model on the indeterminate test set of TBLB specimens is highly promising; however, the main limitation is that the indeterminate test set is small and originated from the same source as the WSIs used to train the model. Further evaluations on test sets of poorly-differentiated TBLB specimens from a variety of sources with varied case distributions are required to validate the model for use in a clinical setting. An incorrect diagnosis resulting from mixing up between ADC and SCC can lead to a patient receiving an inappropriate anticancer treatment. Therefore, it is important that the model is exhaustively validated retrospectively and prospectively. Another limitation of this study is that the model was presented with a constrained diagnostic classification question—classifying between four labels—which is not the same as the diagnostic question typically facing a pathologist when they examine these specimens in clinical practice. In effect, we had excluded cases that had large cell carcinoma, adenosquamous carcinoma, and sarcomatoid carcinoma/carcinosarcoma from this study, while pathologists must consider these tumor types in their differential diagnosis. Pathologists may be performing IHC on indeterminate lung biopsies in part to rule out these entities - if pathologists were posed the same constrained diagnostic classification question as the algorithm, then they might not have felt the need to order IHC to make the distinction.

In addition, we found the performance of SCLC prediction highly promising, where we reserved a larger number of the collected cases for the test set; however, we had the smallest sample size for it compared to ADC and SCC. It is more difficult to collect a larger sample size of SCLC given that the proportion of SCLC cases occurs at approximately less than 10%^29^ in the overall population while NSCLC occurs at approximately 85%^1,30^. None of the SCLC cases we obtained were indeterminate, and they were relatively easy to diagnose by pathologists.

Studies aiming to measure the diagnosis agreement between pathologists for lung carcinomas report highly different ranges, from as low as 0.25 to as high as 0.88 as measured by the kappa statistic^31–36^, with the agreement being typically lower for poorly-differentiated cases. In current clinical workflows, IHC is typically used to attempt to reach a diagnosis when encountering poorly-differentiated cases; however, IHC is not always sufficient (IHC has a reported AUC of 0.94^11^). In those cases, further investigations, such as surgical examinations and computed tomography-guided biopsies, are needed to reach a definitive diagnosis. The integration of an AI model in a clinical workflow to be used instead of IHC or to supplement it as a form of double- or triple-checking would be beneficial in supporting pathologists to reach a diagnosis with shorter delays and fewer errors. Current pathologists’ workflows are not error free; there have been reported cases of errors in diagnosis from small biopsies^37^, as well as cases of delayed diagnosis^38^ and missed diagnosis^39^ in lung cancer. Moreover, there exist several problems regarding pathological diagnosis, such as the shortage of pathologists, disproportion of pathologists between urban and rural areas. These problems may have a negative effect on the quality of the pathological diagnosis and the time needed for the pathological diagnosis. It is the hope that supporting pathologists with AI-assistive tools would be of benefit for both the patients and the pathologists.

For future work, we are planning to validate and refine our model on a large cohort of TBLB and surgical specimens by conducting retrospective and prospective multi-centre studies. We will also attempt to predict genetic alterations and the response of immune checkpoint inhibitor therapy^40^ on HE-stained TBLB and surgical specimens.

## Methods

### Clinical cases and pathological records

For the present retrospective study, we obtained 1,723 cases (1,223 TBLB and 500 surgical specimens) of human pulmonary lesions H&E-stained histopathological specimens from the surgical pathology files of Kyushu Medical Center after histopathological review of those specimens by surgical pathologists. In addition, we obtained 500 cases of human pulmonary lesions HE-stained surgical specimens from International University of Health and Welfare, Mita Hospital (Tokyo) after histopathological review and approval by surgical pathologists. Pathological records, including histopathological definitive diagnoses for TBLB and surgical specimens, and immunohistochemical results for a subset of TBLB specimens were analyzed in this study. Only cases that either had ADC, SCC, SCLC, or non-neoplastic (any tissue that does not contain tumour cells) were included, and any case that had large cell carcinoma, adenosquamous carcinoma, sarcomatoid carcinoma/carcinosarcoma, pulmonary blastoma, and endodermal tumour were excluded. The experimental protocols were approved by the Institutional Review Board (IRB) of the Kyushu Medical Center (No. 20C036) and International University of Health and Welfare (No. 19-Im-007). All research activities complied with all relevant ethical regulations and were performed in accordance with relevant guidelines and regulations in Kyushu Medical Center and International University of Health and Welfare, Mita Hospital. Informed consent to use histopathological samples and pathological diagnostic reports for research purposes had previously been obtained from all patients prior to the surgical procedures at both hospitals, and the opportunity for refusal to participate in research had been guaranteed by an opt-out manner. The test cases were selected randomly, so the ratio of carcinomas (ADC, SCC, and SCLC) to non-neoplastic cases in test sets was reflective of the case distributions at the providing institutions. All WSIs from both Kyushu Medical Center and Mita were scanned at a magnification of x20.

Among specific IHC markers for pulmonary epithelium, thyroid transcription factor-1 (TTF1) is the most widely used. Up to 94% of pulmonary ADC have been reported to express TTF1^41^, while TTF1 is rarely or only minimally expressed by poorly differentiated SCC^42^. Studies have shown that most poorly differentiated SCC will stain for p63, whereas SCLCs demonstrate the opposite pattern of immunoreactivities (p63-negative, high molecular weight keratin-negative, and TTF1-positive)^41,42^. Staining of ADC is more variable. Similarly, most poorly differentiated SCC will stain for high molecular weight keratin, whereas SCLCs will not stain^42^. Moreover, TTF1 (ADC marker), and p40 (SCC marker), have been reported to be the most precise differential diagnostic markers to discriminate carcinoma subtypes^43,44^.

A surgical section is excised from a surgically-resected lung specimen. To address cancer characteristics (e.g., size, location within lobe and segment, relation with bronchi, extension to pleura) precisely, usually several sections (e.g., tumour sections including one showing relationship to bronchus, non-neoplastic lung sections, and bronchial line of cross-sections) are examined for pathological diagnoses on routine practical surgical resection specimens^45^. This is what can be seen in Fig. 5.

### Dataset and annotations

The datasets obtained from Kyushu Medical Centre and International University of Health and Welfare, Mita Hospital, consisted of 1,723 and 500 WSIs, respectively. In addition, we used a total of 905 WSIs from The Cancer Genome Atlas (TCGA) ADC and SCC projects (TCGA-LUAD and TCGA-LUSC). The pathologists excluded cases from those projects that were inappropriate or of poor quality for this study. The diagnosis of each WSI was verified by two pathologists specialized in pulmonary pathology. Table 2 breaks down the distribution of the datasets into training, validation, and test sets. The training set was solely composed of WSIs of TBLB specimens. The patients’ records were used to extract the WSIs’ pathological diagnoses. The final diagnoses were either reached by visual inspection of the HE-stained slides or by further investigations via immunohistochemical and/or histopathological examination of the surgically resected tumour samples. Cases that had further investigations performed were placed into the indeterminate subsets. 215 TBLB WSIs from the training and validation sets had a neoplastic diagnosis (122 ADCs, 72 SCCs, and 21 SCLCs). They were manually annotated by a group of three surgical pathologists who perform routine histopathological diagnoses. The pathologists carried out detailed cellular-level annotations by free-hand drawing around well-differentiated and poorly-differentiated carcinoma cells that corresponded to ADC, SCC, or SCLC. The specimens in the indeterminate sets were exclusively composed of poorly-differentiated carcinoma cells where it was difficult to distinguish between ADC and SCC. Poorly-differentiated does not always imply indeterminate as some poorly-differentiated cases can be distinguished. The indeterminate sets did not contains such cases. The rest of the sets contained either well-differentiated or a mix of poorly- and well-differentiated carcinoma cells within the same slide. The non-neoplastic subset of the training and validation sets (423 WSIs) was not annotated and the entire tissue areas within the WSIs were used. Each annotated WSI was observed by at least two pathologists, with the final checking and verification performed by a senior pathologist.

### Deep learning model

Our deep learning model consisted of two separately-trained components: a CNN tile classifier and an RNN tile aggregator for WSI diagnosis.

To apply the CNN classifier on the WSIs, we performed slide tiling, where a given WSI at a magnification of x10 was divided into overlapping tiles of 224x224 pixels, with a stride of 112x112 pixels. We only used tiles from the tissue regions, and we did this by performing a thresholding on a grayscale WSI using Otsu’s method^46^, which eliminated most of the white background which typically occupies a large proportion of the biopsy WSI. We then sampled tiles from the WSIs for training. If the WSI contained any cancer cells, then we only sampled from the annotated regions such that the centre point of the tile was within the annotation region. We did not sample tiles from unannotated regions of WSIs with cancer, as their label could still be ambiguous given that that the pathologists did not exhaustively annotate each WSI. On the other hand, if the WSI did not contain cancer cells (non-neoplastic), then we freely sampled from the WSI.

For the CNN tile classifier, we used the EfficientNet-B1^47^ architecture with a global-average pooling layer followed by a softmax classification layer with four outputs: ADC, SCC, SCLC, and non-neoplastic.

During testing, we apply the model on the entire tissue within a WSI. We did this by tiling the tissue areas, and then feeding all the tiles into the CNN tile classifier resulting in four output probabilities per tile. WSI diagnosis probabilities can be obtained by using a simple max-pooling approach where for each label the WSI probability is computed as the maximum of the tile probabilities; however, such an approach is more prone to yielding false positives in some cases, as all it would take is a single false positive tile to alter the WSI diagnosis. A WSI can have more than one label predicted if the probability for that label is larger than the threshold (typically 0.5).

We trained the CNN model using fully-supervised learning and transfer learning. We used balanced sampling of tiles to ensure that there was equal representation of all labels in a training batch, given that the training set was imbalanced (see Table 2). We did this by having four queues, where each held the WSIs of each label. We then went in turn randomly sampling $\frac{\text {batch size}}{4}$ tiles from each queue to form a single batch. Whenever a queue reached its end, it was repopulated and reshuffled. An epoch ended when all the WSI were at least seen once.

In addition, we performed data augmentation by randomly transforming the input tiles with flipping, 90 degree rotations, and shifts in brightness, contrast, hue, and saturation. We used the categorical cross entropy loss function, and we trained the model with the Adam optimisation algorithm^48^ with the following parameters: $beta_1=0.9$, $beta_2=0.999$, batch size of 32, and a learning rate of 0.001 with a decay of 0.95 every 2 epochs. We tracked the performance of the model on a validation set, and the model with the lowest loss on the validation set was chosen as the final model. The CNN model weights were initialised with pre-trained weights on ImageNet^49^, and for the first epoch we froze all the base layers and only trained the final classification layer. After the first epoch, all the weights were unfrozen, allowing them to train.

For the RNN model, we used an architecture with a single hidden layer of a gated recurrent unit (GRU)^50^ with a size of 128 followed by a classification layer with a sigmoid activation and three outputs (ADC, SCC, and SCLC). The inputs to the RNN model were the feature representation vectors obtained from the global-average pooling layer of the CNN classifier. All the feature vectors were fed into the RNN model to obtain the final WSI diagnosis. We trained the RNN model after the CNN model finished training. We extracted a set of feature vectors for all the WSIs in the training set using the CNN model as a feature extractor. Each WSI had a variable number of feature vectors and an associated WSI diagnosis. We trained the model with the Adam optimisation algorithm, with similar hyperparameters as the CNN training, except with a batch size of one.

### Software and statistical analysis

We implemented the deep learning model using TensorFlow^51^. We calculated the AUCs and log losses in python using the scikit-learn package^52^ and performed the plotting using matplotlib^53^. We performed image processing, such as the thresholding with scikit-image^54^. We computed the 95% CIs estimates using the bootstrap method^55^ with 1000 iterations. We used openslide^56^ to perform real-time slide tiling.

## Data Availability

Due to specific institutional requirements governing privacy protection, the majority of datasets used in this study are not publicly available. The external lung TCGA (TCGA-LUAD and TCGA-LUSC project) is publicly available through the Genomic Data Commons Data Portal (https://portal.gdc.cancer.gov/).
